# Facile Fabrication of Polyvinyl Alcohol/Edge-Selectively Oxidized Graphene Composite Fibers

**DOI:** 10.3390/ma12213525

**Published:** 2019-10-28

**Authors:** Taehoon Kim, Gayeong Han, Yeonsu Jung

**Affiliations:** Composites Research Division, Korea Institute of Materials Science, Changwon 51508, Korea; hangayeong@kims.re.kr (G.H.); ysjung@kims.re.kr (Y.J.)

**Keywords:** PVA fiber, graphene, EOG, composite fiber, wet spinning

## Abstract

Graphene derivatives are effective nanofillers for the enhancement of the matrix mechanical properties; nonetheless, graphene oxide (GO), reduced GO, and exfoliated graphene all present distinct advantages and disadvantages. In this study, polyvinyl alcohol (PVA) composite fibers have been prepared using a recently reported graphene derivative, i.e., edge-selectively oxidized graphene (EOG). The PVA/EOG composite fibers were simply fabricated via conventional wet-spinning methods; thus, they can be produced at the commercial level. X-ray diffractometry, scanning electron microscopy, and two-dimensional wide-angle X-ray scattering analyses were conducted to evaluate the EOG dispersibility and alignment in the PVA matrix. The tensile strength of the PVA/EOG composite fibers was 631.4 MPa at an EOG concentration of 0.3 wt %, which is 31.4% higher compared with PVA-only fibers (480.6 MPa); compared with PVA composite fibers made with GO, which is the most famous water-dispersible graphene derivative, the proposed PVA/EOG ones exhibited about 10% higher tensile strength. Therefore, EOG can be considered an effective nanofiller to enhance the strength of PVA fibers without additional thermal or chemical reduction processes.

## 1. Introduction

The ultrahigh tensile strength (~130 GPa) and Young’s modulus (~1 TPa) of graphene [1] make it an excellent nanofiller to enhance the mechanical properties of composites [2]; however, its poor dispersibility prevents the preparation of high-strength composites. Thus, graphene oxide (GO) can be used as an alternative because of the high dispersibility induced by its oxygen functional groups, including hydroxyl, epoxide, and carboxyl groups [2,3,4,5]. Indeed, GO has improved the mechanical properties of epoxy [6], poly(L-lactic acid) [7], polycarbonate [8], and polyimide [9,10].

Polyvinyl alcohol (PVA) fibers present high mechanical strength, non-toxicity, biocompatibility, and environmental-friendly fabrication process [11]. Nonetheless, their tensile strength is somewhat lower than those of aramid and poly(p-phenylene benzobisoxazole) fibers and, thus, many researchers have attempted the increase it [12,13]. A way to improve the mechanical properties is the preparation of nanocomposite fibers by incorporating high-strength nanofillers, such as graphene derivatives, into PVA fibers [14,15]. PVA/GO composite fibers have been widely investigated because both their components have oxygen functional groups, resulting in good compatibility between them; indeed, they possess enhanced tensile strength compared with PVA-only fibers [16,17,18]. However, GO has intrinsically poorer mechanical properties than pure graphene due to its many defects and the destruction of the sp2 bonding between carbon atoms during the oxidation process [19]. To further increase the strength of composite fibers by using non-oxidized graphene, some research groups have replaced GO with reduced GO (rGO); since the GO reduction restores the sp2 bonding, rGO has higher mechanical properties than GO [20]. Unfortunately, the relatively low dispersibility of rGO decreases the processability of its solutions and may lead to the formation of aggregation and defect sites in the composite fibers. High strength and dispersibility of graphene-based nanofillers are required simultaneously but are hard to achieve.

In a previous work, we synthesized edge-selectively oxidized graphene (EOG), attaining simultaneous high dispersibility and electrical properties [21]. Since the graphene edge is selectively oxidized, the sp2 bonding on its basal plane is preserved, unlike in GO. The presence of carboxyl groups on the EOG edge enables dispersion in aqueous systems and solution processability. Therefore, EOG can be the ideal high-strength and high-dispersibility graphene nanofiller but, to the best of our knowledge, no studies have been conducted on PVA/EOG composite fibers.

Herein, for the first time, we reported PVA/EOG composite fibers having enhanced tensile strength by adding an EOG solution to the spinning solution. In this paper, we first described the preparation method for the PVA/EOG composite fibers, which can be prepared using the same method used for preparing PVA fibers. Second, the structural and morphological features of the composite fibers were analyzed to observe the dispersion state of the EOG. Lastly, we compared the tensile strength of the PVA/EOG composite fibers, PVA/GO composite fibers, and PVA-only fibers to analyze the effects of EOG as a reinforcement.

## 2. Materials and Methods

### 2.1. Materials

Natural graphite, potassium persulfate (98%), phosphorus pentoxide (98%), potassium permanganate (98%), PVA (M_W_ = 146,000–186,000, 99+% hydrolyzed), and dimethyl sulfoxide (DMSO) were purchased from Sigma Aldrich. Sulfuric acid (98%), hydrogen peroxide (30%), hydrochloric acid (35%–37%), and methanol (99.5%) were provided by Samchun Chemical. All chemicals were used without further purification. EOG and GO were synthesized based on our previously reported methods [3,4,21] and prepared as colloidal suspensions with various concentrations (up to 12 mg/mL). The size of the EOG and GO was 2.5 um and 25 μm, respectively. To confirm the effect of dispersion of EOG in PVA matrix, liquid exfoliated graphene was also synthesized according to our previous works [21] and dispersed in distilled water, with a concentration of 1.2 mg/mL.

### 2.2. Preparation of PVA-Only and PVA Composite Fibers

PVA (1.0 g) was dissolved in 10 mL of DMSO at 80 °C under strong agitation overnight. To prepare the spinning dope solutions for the PVA-only, PVA/EOG composite, PVA/GO composite, and PVA/exfoliated graphene composite fibers, 2.5 mL of distilled water, EOG suspension, GO suspension, and liquid exfoliated graphene suspension, respectively, were added to the corresponding PVA solutions. The concentrations of the EOG and GO suspensions were set so to obtain composite fibers with a filler concentration range of 0.1–3.0 wt %.

The spinning dope solutions were transferred into a syringe and spun into a coagulation bath that contained 100% methanol through 18-gauge metal needles. The resulting spun solution was immediately turned into gel fibers, which were coagulated for 1 h at room temperature, dried at 50 °C in a vacuum oven for 3 h, and, finally, drawn at 150 °C in air at a drawing ratio of 8. The spinning process is a batch process. This method was adopted for all the types of composite fibers investigated.

### 2.3. Characterization

X-ray diffractometry (XRD) analysis was conducted on the PVA-only and various PVA composite fibers in the reflection mode, which is a standard procedure for XRD measurements, by using a D/Max 2500 diffractometer (Rigaku) with Ni-filtered Cu Kα radiation (λf = 0.154184 nm)). Scanning electron microscopy (SEM) cross-section images of the fabricated fibers were obtained with an SEM system (SNE-4500M plus, SEC) at 15.0 kV. Two-dimensional (2D) wide-angle X-ray scattering (WAXS) patterns were recorded by a D8 discover (Bruker) with Cu Kα radiation (λf = 0.154184 nm). The mechanical properties were investigated by using a universal testing machine (3344, Instron) according to the ASTM standard C1557 with a 5 N load cell; the gauge length of the fiber sample was 1 cm and the crosshead speed was 0.2 mm/min. The cross-sectional area of the fibers is characterized by calculations of linear density and confirmed by SEM analysis. The diameter of the fibers was about 55–63 μm after drawing and about 170–180 μm before drawing. We performed tensile tests for 15 samples for each fiber to minimize the errors of the results.

## 3. Results and Discussion

**Figure 1:**
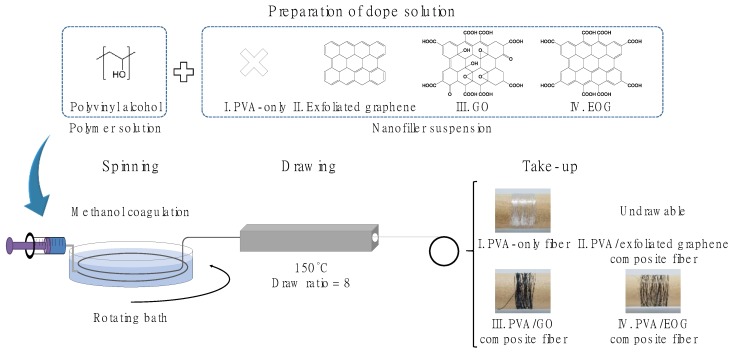
Schematic representation of the spinning dope solution preparation, spinning, and drawing processes carried out in this work.

Figure 1 schematizes the preparation of the PVA/EOG composite fibers. All processes of preparation of the PVA/EOG fibers are basically the same as for the PVA spinning except for the EOG addition into the spinning solution, which means that there is no need for additional processing nor changing of the fabrication parameters. No aggregation sites were observed during the preparation and spinning of the PVA/EOG dope solution, indicating a stable dispersion of EOG. The PVA/GO composite fibers were successfully spun and drawn based on the same method, which is a reasonable result considering the high dispersibility of GO. In the case of PVA/exfoliated graphene composite fibers, the solution was still successfully spun but the resulting fibers were broken during the drawing process; the poor dispersibility of the exfoliated graphene presumably decreased the fiber drawability because the aggregated graphene acted as defect sites. Therefore, in this work, PVA/exfoliated graphene composite fibers were disregarded for the further discussions of this study, and PVA/EOG and PVA/GO composite fibers were characterized to compare the effect of different graphene derivatives.

**Figure 2:**
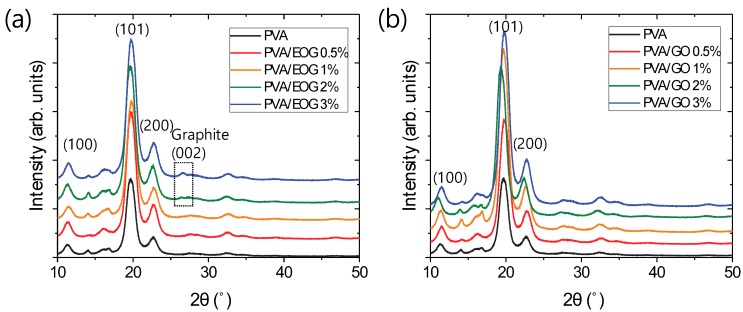
Comparison of the X-ray diffraction profiles of polyvinyl alcohol (PVA)-only fibers with those of (**a**) PVA/edge-selectively oxidized graphene (EOG) and (**b**) PVA/graphene oxide (GO) composite fibers.

The structure of the PVA-only and PVA/EOG composite fibers after the hot-drawing process was characterized by XRD to examine the crystallinity of the PVA fibers and restacked graphene derivatives (Figure 2a). The PVA-only fibers and PVA composite fibers prepared in this work exhibited high crystallinity compared with PVA films [22] because they were highly drawn. The XRD peaks at about 11.4°, 19.7°, and 22.7° were assigned to the crystal faces of (100), (101), and (200), respectively [23,24]. The XRD patterns of the PVA/EOG composite fibers were similar to that of the PVA-only ones, indicating that the EOG incorporation did not decrease the fiber crystallinity. A small peak of the (002) graphite plane (~26°) appeared at the EOG concentration of 2.0 wt % and 3.0 wt %, which is likely too high/inappropriate for enhancing the mechanical properties of the composite fibers. On the other hand, the peak was not observed when the EOG concentration was below 1.0 wt %. These results suggest that EOG was well dispersed in the PVA matrix with no aggregation sites at least at the resolution level of the instrument. Further information on the aggregation and dispersion in this concentration range can be deduced from the mechanical properties of the PVA/EOG composite fibers. In the case of PVA/GO composite fibers (Figure 2b), unfortunately, the dispersion state of GO could not be determined via XRD because its (100) peak lays close to the PVA (100) one and, therefore, a comparison with the EOG dispersion was not possible.

**Figure 3:**
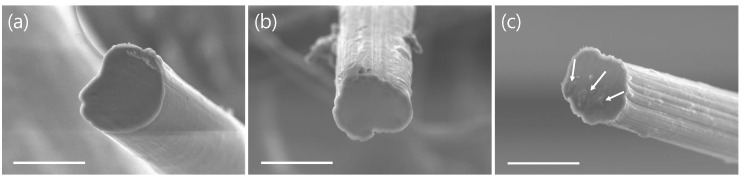
Scanning electron microscopy cross-section images of polyvinyl alcohol composite fibers with (**a**) 0.5, (**b**) 1.0, and (**c**) 3.0 wt % edge-selectively oxidized graphene; the scale bar is 50 um.

To visually evaluate the dispersion state of EOG in the PVA matrix, the cross-sections of the PVA/EOG composite fibers were observed by SEM (Figure 3); the almost circular shape of the fiber cross-sections indicates that the spinning, gelation, and hot-drawing processes were performed properly. The cross sections were smooth for low EOG concentrations, indicating that EOG was well dispersed in the PVA matrix, in agreement with the XRD results. However, some aggregation sites were observed when the EOG concentration was 3.0 wt %; this result, also well correlated with the XRD analysis, suggests that a high EOG concentration may decrease the mechanical properties of the composite fibers.

**Figure 4:**
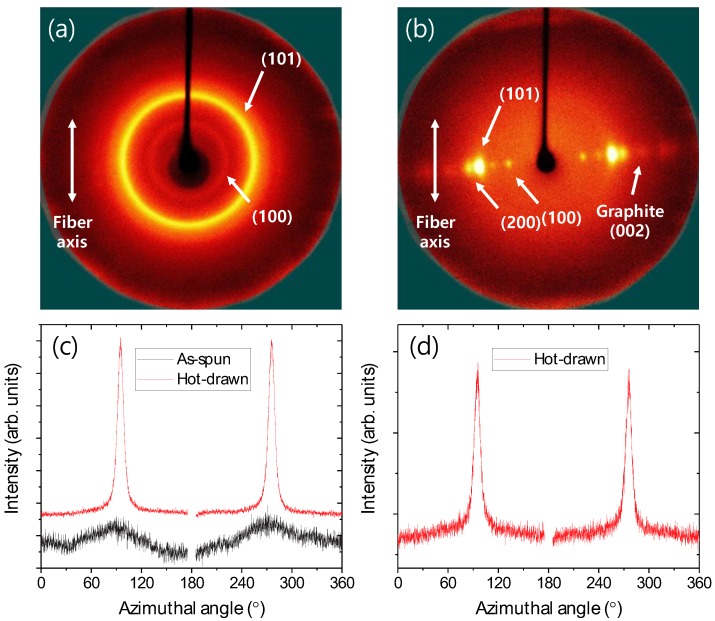
Two-dimensional wide-angle X-ray scattering patterns of (**a**) as-spun (**a**) and (**b**) hot-drawn polyvinyl alcohol/(3.0 wt %) edge-selectively oxidized graphene composite fibers and their angular scattered intensity at 2θ of (**c**) 19°–23° and (**d**) 26°–27°.

Two-dimensional WAXS patterns of PVA/EOG composite fibers with 3.0 wt % EOG were examined to verify the alignment of PVA and EOG; both the as-spun and hot-drawn fibers were analyzed so to evaluate the effect of the drawing process (Figure 4). Although the as-spun fibers possessed crystallinity, the (100) and (101) planes exhibited an isotropic broad ring pattern, which means that the crystalline sites had no directional alignment. However, after drawing, the 2D WAXS patterns became clearer and that from the (200) plane was also observed; they were located in the equatorial region, indicating that the PVA chains were highly aligned along the fiber axis. Moreover, the (002) graphite 2D WAXS pattern also appeared after drawing, which suggests that EOG was also well aligned along the PVA fiber.

**Figure 5:**
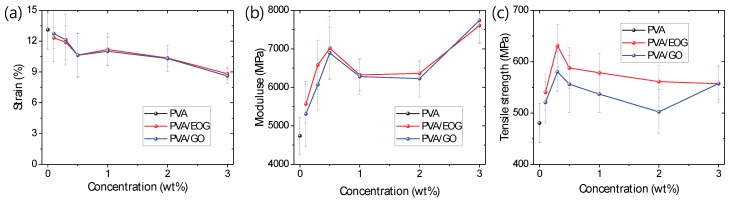
(**a**) Tensile strain, (**b**) Young’s modulus, and (**c**) tensile strength of polyvinyl alcohol (PVA)-only (black), PVA/edge-selectively oxidized graphene (EOG) composite (red), and PVA/graphene oxide (GO) composite fibers (blue).

The tensile strength of the hot-drawn PVA-only and PVA/EOG composite fibers were measured. Table 1 and Figure 5 summarize the mechanical properties of the samples, showing that even small amounts of EOG significantly improves the tensile strength and Young’s modulus of the fibers. The tensile strength and Young’s modulus of the composite fibers increased linearly to a 0.3 wt % concentration in proportion to the EOG content. The composite fibers reached the highest tensile strength (631.4 MPa) when the EOG concentration was 0.3 wt %, which is an increase of 31.4% compared to the PVA-only fibers (480.6 MPa). Therefore, the PVA/EOG composite fibers follow the rule of mixture up to the concentration of 0.3 wt %. When the EOG concentration exceeds 0.3 wt %, even though the XRD and SEM analyses did not reveal EOG aggregations for concentrations up to 2.0 wt %, the tensile strength of the composite fibers no longer follow a linear increase. The addition of large amounts of EOG apparently decreased Young’s modulus as well as tensile strength. It is believed that the aggregated EOG resulted in a reduction of interfacial area, stress transfer, and alignment of EOG nanofiller [12]. Therefore, addition of the appropriate amount of EOG into PVA fiber is crucial to obtain composite fibers of optimized mechanical properties. With respect to other fillers, graphene derivatives exhibit the highest tensile strength at low filler concentrations [14], which demonstrates their high effectiveness for strength enhancement.

To evaluate the performance of EOG as a nanofiller, the mechanical properties of the PVA/GO composite fibers were also measured for comparison (Figure 5). The PVA/EOG and PVA/GO composite fibers showed similar trends in the strain at break. Similarities were observed also as regards the Young’s modulus, but the PVA/EOG composite fibers exhibited higher values; in particular, they showed values 4.9% and 8.5% higher than those of the PVA/GO composite fibers for concentrations of 0.1 wt % and 0.3 wt %, respectively. However, the two types of composite fibers differed greatly in terms of tensile strength. They both showed the highest tensile strength at a filler concentration of 0.3 wt %, and this maximum value for the PVA/GO composite fibers (580.4 MPa) was about 10% lower than that of the PVA/EOG ones. Therefore, the incorporation of EOG rather than GO as a nanofiller can more effectively improve the mechanical properties of PVA fibers.

## 4. Conclusions

We fabricated PVA/EOG composite fibers with high crystallization, alignment, no aggregation site, and improved strength. The preparation method was essentially the same as that for PVA-only fibers apart from the addition of an EOG solution instead of distilled water. This means that the composite fibers can be produced in current commercial facilities because there are no additional processes such as thermal or chemical reduction processes. The tensile strength of the obtained PVA/EOG composite fibers increased by 30% with only the addition of 0.3wt % EOG. This work proves that EOG, which has a sp2 conjugation and high dispersibility simultaneously, is a better reinforcing nanofiller than GO and exfoliated graphene, which are conventional graphene nanofillers.

In this study, our research is limited in the preparation of high-strength fibers based on a new graphene derivative. Further research work should focus on maximizing the strength of the composite fibers through additional functionalization of EOG and crosslinking between EOG and PVA, and investigation of high strength conductive PVA/EOG composite fibers is required.

## Figures and Tables

**Table 1:** Mechanical properties of polyvinyl alcohol (PVA)-only and PVA/edge-selectively oxidized graphene composite fibers, after hot-drawing.

Concentration (wt %)	Tensile Strength (MPa)	Young’s Modulus (GPa)	Tensile Strain (%)
0	480.6	4.74	13.1
0.1	540.4	5.57	12.3
0.3	631.4	6.59	11.9
0.5	587.9	7.02	10.6
1	578.4	6.32	11.2
2	561.2	6.36	10.4
3	557.1	7.61	8.8
